# Supplementary material for: Phosphorylation-dependent pseudokinase domain dimerization drives full-length MLKL oligomerization
Source: Nat Commun. 2023 Oct 26;14:6804. doi: 10.1038/s41467-023-42255-w (PMC10603135; doi:10.1038/s41467-023-42255-w)
Supplement: Supplementary file 1 — Supplementary Information [file 41467_2023_42255_MOESM1_ESM.pdf]

Supplementary Table 1. X-ray crystallography data collection and refinement statistics

**Structural parameters**	**Human p-MLKL pseudokinase domain (pT357/pS358, residue 190-471)** (PDB: 8SLZ)
**Data Collection Statistics**	
Wavelength (Å)	0.9537
Resolution range (Å)	47.72 - 2.30 (2.386 - 2.304)[a]
Space group	C *2 2 2$_1$*
Unit cell (x, y, z, a, b, g)	70.811 74.961 127.227 90 90 90
Total reflections	100968 (9942)
Unique reflections	15077 (1427)
Multiplicity	6.7 (6.9)
Completeness (spherical) (%)	98.09 (94.94)
Mean I/sigma(I)	16.21 (1.76)
Wilson B-factor	51.29
R-merge	0.0711 (0.9642)
R-meas	0.07727 (1.042)
R-pim	0.02981 (0.3922)
CC1/2	0.999 (0.748)
CC*	1 (0.925)
**Refinement Statistics**	
Reflections used in refinement	15052 (1427)
Reflections used for R-free	1507 (149)
R-work	0.2002 (0.2741)
R-free	0.2340 (0.3103)
Number of non-hydrogen	2134
macromolecules	2099
solvent	35
Protein residues	265
RMS(bonds)	0.007
RMS(angles)	0.7
Ramachandran favored (%)	98.85
Ramachandran allowed (%)	1.15
Ramachandran outliers (%)	0
Rotamer outliers (%)	3.08
Clashscore	1.66
Average B-factor	57.83
macromolecules	57.88
solvent	54.90

[a]Statistics for the highest-resolution shell are shown in parentheses

Supplementary Table 2 | Oligonucleotide sequences

hRIPK3 316 stop KpnI rev‡	5′- CTCCCCCATCTCCCGTTAagaaaatctcctattgctgctcctgag-3′
NcoI RBS hRIPK3 C3S fwd	5′- GGATCTCGAGCCATGGAAACCATGtcgTCCgtcaagttatggccc-3′
pFB_HTb AccI fwd	5′-AGCTATAGTTCTAGTGGTTGGCTACGT-3′
pFB_HTb AccI rev	5′-AGAGTCCTGGGCGAACAAACG-3′
pAB2 AccI polyA fwd	5′-TTCGCCCAGGACTCTCCCCGCGTTTATGAACAAACG-3′
pAB2 p10 rev	5′-ACTAGAACTATAGCTCGGACCTTTAATTCAACCCAACACAA-3′
Monobody BamHI fwd	5′- CGCGGATCCGTTTCTTCTGTTCCGACCAAAC-3′
Monobody stop XbaI rev	5′- agttactagTCTAGATTAggtacggtagttaatcgagattg-3′
NcoI RBS Monobody fwd	5′- ggatctcgagccatggaaaccatgggatccgtttcttctgttcc -3′
Monobody stop KpnI rev	5′- CTCCCCCATCTCCCGTTAggtacggtagttaatcgagattgGGCT -3′
hMLKL C184S fwd	5′- gtatttaccaccaaaaAgcatgcaggagatc-3′
hMLKL C184S rev	5′- gatctcctgcatgcTttttggtggtaaatac-3′
hMLKL 2 BamHI fwd	5′- CGCggatccgaaaatttgaagcatattatc -3′
hMLKL 190 BamHI fwd	5′- CGCGGATCCcaagagcaaatcaaggagatcaag-3′
hMLKL RBS BamHI fwd	5′- CGCGGATCCGCGCCACCATGgaaaatttgaagcatattatcaccc -3′
hMLKL 471 stop EcoRI rev	5′- CGCGAATTcacttagaaaaggtggagagtttc-3′
hMLKL 154 stop EcoRI rev	5′- CGCGAATTCAatctcttcttagcatctggaaagc-3′
HRV3C hMLKL 130 fwd	5′- agttctgttccaggggcccGGAGCGTCCTGGGCACA -3′
hMLKL 123 HRV3C rev	5′- ccctggaacagaacttccagAGGCATGCGTTGCTCAAC -3′

‡ Restriction sites underlined

Supplementary Table 3. Unit cell dimensions for structure reported in Table 2.

Identifier	Structure Data						
**Entry ID**	**Length a (Å)**	**Length b (Å)**	**Length c (Å)**	**Angle α (°)**	**Angle β (°)**	**Angle γ (°)**	**Space Group**
4MWI	72.114	74.723	127.601	90	90	90	C *2 2 2_1*
8SLZ	70.811	74.961	127.227	90	90	90	C *2 2 2_1*
4M67	71.499	75.12	126.817	90	90	90	C *2 2 2_1*
6LK6	70.821	75.709	128.494	90	90	90	C *2 2 2_1*
6LK5	71.739	74.848	127.388	90	90	90	C *2 2 2_1*
6BWK	72.114	74.723	127.601	90	90	90	C *2 2 2_1*
5KO1	71.4	74.88	127.07	90	90	90	C *2 2 2_1*
5KNJ	54.488	90.879	115.618	90	90	90	P *2_1 2_1 2_1*
6O5Z	50.161	118.845	52.761	90	116.78	90	P *2_1*
7JW7	64.805	64.805	226.763	90	90	120	P *3_1 2 1*
7JXU	94.619	94.619	115.197	90	90	90	P *4_3*
7MON	64.495	82.839	104.842	90	90	90	P *2_1 2_1 2_1*
4BTF	42.4	78.3	162.938	90	90	90	P *2_1 2_1 2_1*
4M69	106.443	141.951	107.129	90	90	90	C *2 2 2_1*
4M68	41.97	61.522	60.074	90	100.29	90	P *2_1*
6VC0	155.532	124.775	81.053	90	112.75	90	C *2*
6VBZ	190.057	190.057	77.759	90	90	120	P *6_1 2 2*

Supplementary Figure 1. Generation of recombinant p-MLKL pseudokinase domain.

a. Size-exclusion chromatogram of p-MLKL pseudokinase domain (residue 190-471; black line) overlaid with unphosphorylated, wild-type (apo) human MLKL pseudokinase domain (red dashed line). Fractions used for intact protein mass spectrometry experiment are labelled on the x-axis. **b.** Intact protein mass spectra (MS) of p-MLKL pseudokinase domain size-exclusion chromatography fractions and the unphosphorylated human MLKL pseudokinase domain. **c.** Relative intensities of phosphorylated residues in a tryptic digest MS/MS of p-MLKL and apo MLKL pseudokinase domain. **d.** MS/MS spectrum of the phosphopeptide LAGFELRKTQTSMSLGTTR (MLKL pT355/pS358/pS360). **e.** MS/MS spectrum of the phosphopeptide KTQTSMLGTTR (MLKL pT357). **f.** Recombinant p-MLKL pseudokinase domain (2 mg/mL) dephosphorylated with recombinant lambda phosphatase (0.4 mg/mL) eluted from a Superdex 200 10/300 Increase column (Cytiva) (dashed red line) at the same retention time as monomeric, unphosphorylated MLKL pseudokinase domain (black solid line). Elution volumes of gel filtration standards (17-670 kDa) are annotated above the chromatograms.

Supplementary Figure 1

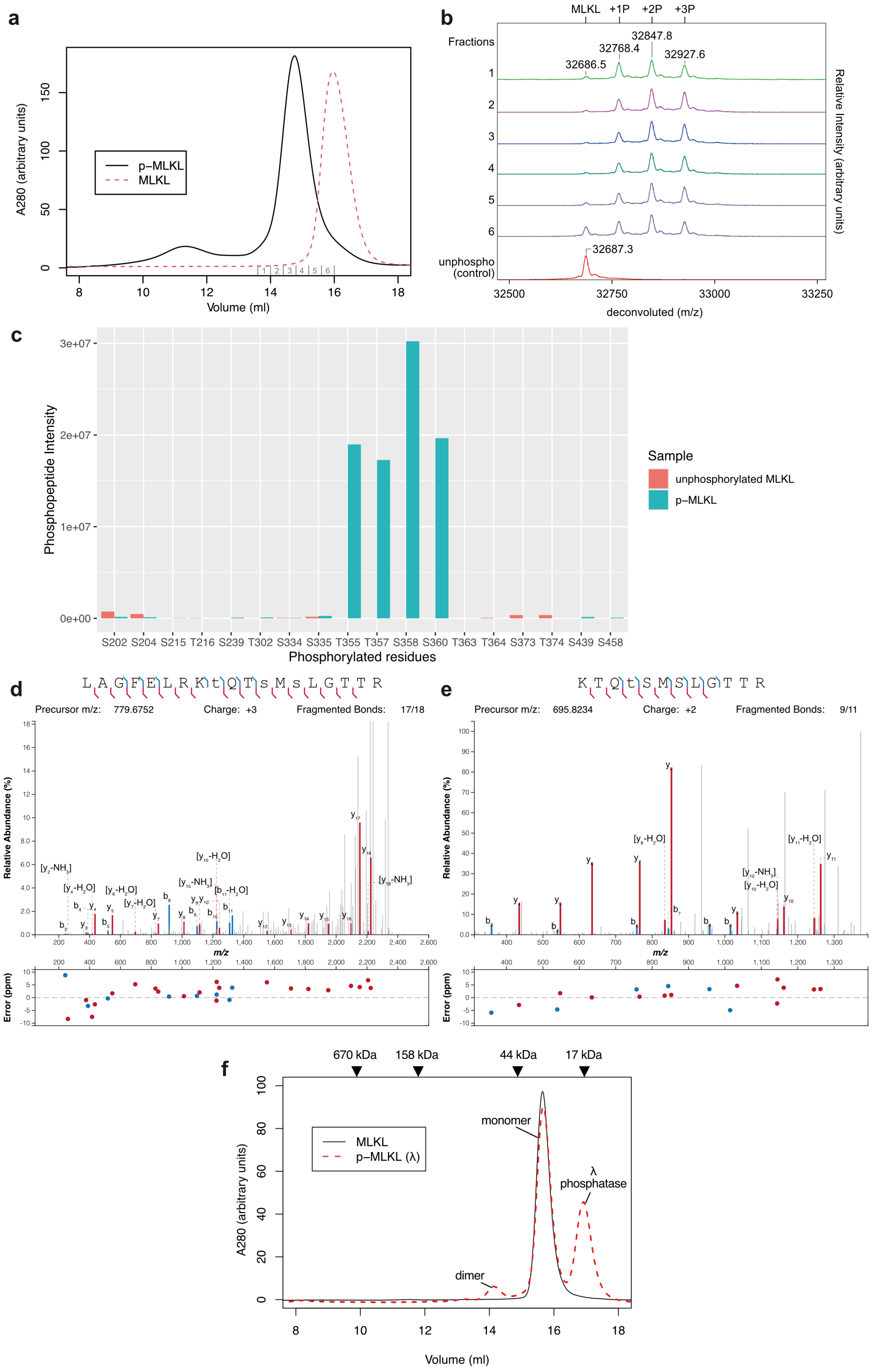

Supplementary Figure 2. RIPK3-mediated phosphorylation drives human MLKL pseudokinase domain dimerization by favoring the closed conformation.

a-b. Human MLKL pseudokinase domain (grey ribbon; yellow, αC helix; green, activation loop) undergoes a conformational change upon phosphorylation of the activation loop (T357/S358). **a**. Crystal structure of p-MLKL pseudokinase domain adopt the closed conformation with aligned R-spine (sticks and transparent surface). **b.** The previously published structure of MLKL pseudokinase domain in complex with RIPK3 kinase domain[1] adopts the open conformation with disrupted R-spine (sticks and transparent surface). **c**. MLKL pseudokinase domain dimerizes in the crystal lattice of p-MLKL in a closed conformation (bottom), but the same dimerization interface does not exist in the lattice of MLKL:RIPK3 co-crystal structure where MLKL is in an open conformation (top).

Supplementary Figure 2

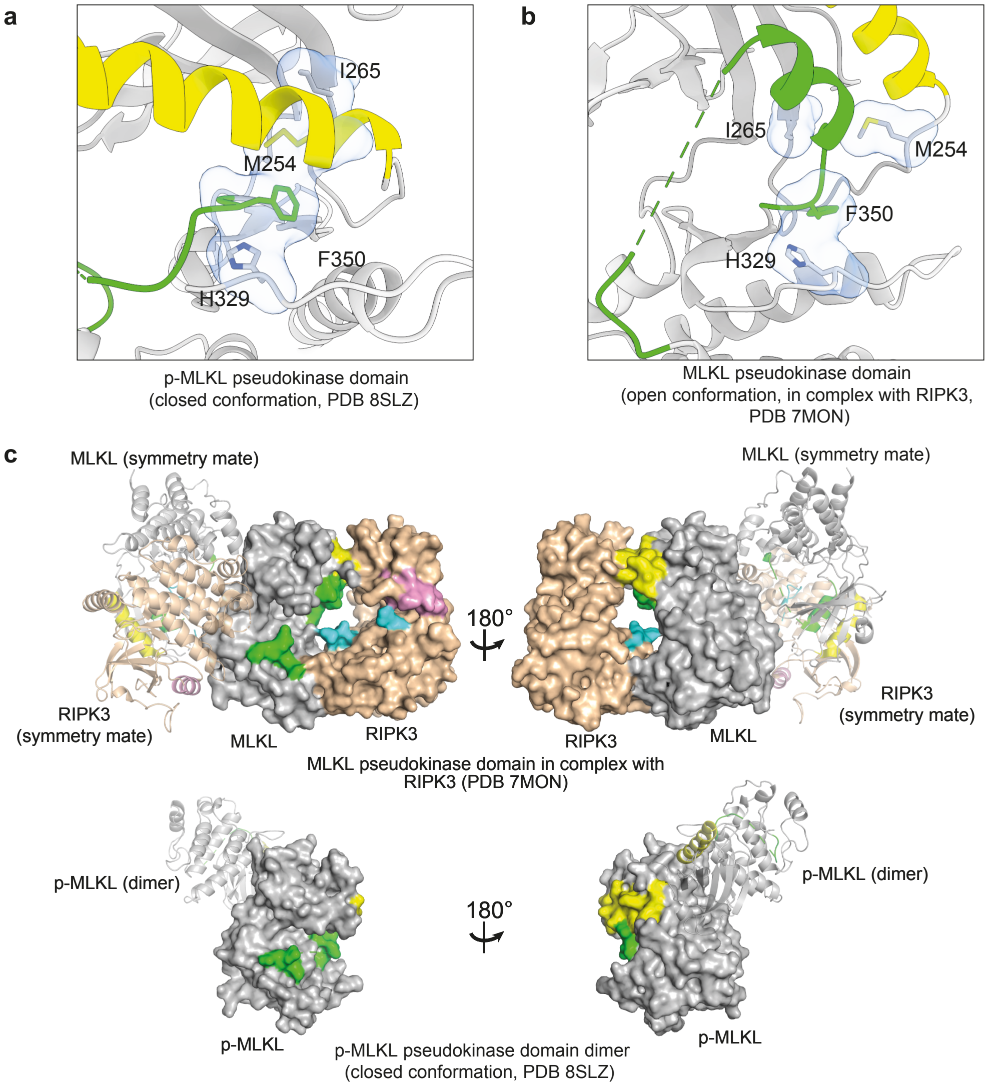

Supplementary Figure 3. Intermolecular disulfide bonds within human MLKL tetramer are mediated by C184.

a. Annotated MS/MS spectra of homeotypic disulfide-linked peptides from the tryptic digest of human MLKL tetramer (from co-expression with RIPK3 kinase domain). **b**. The model of human MLKL tetramer predicts C184 to be on a disordered loop between the pseudokinase domain and the brace helix coiled coil. Disulfide bond formation may bring these loops closer than those in this structural model.

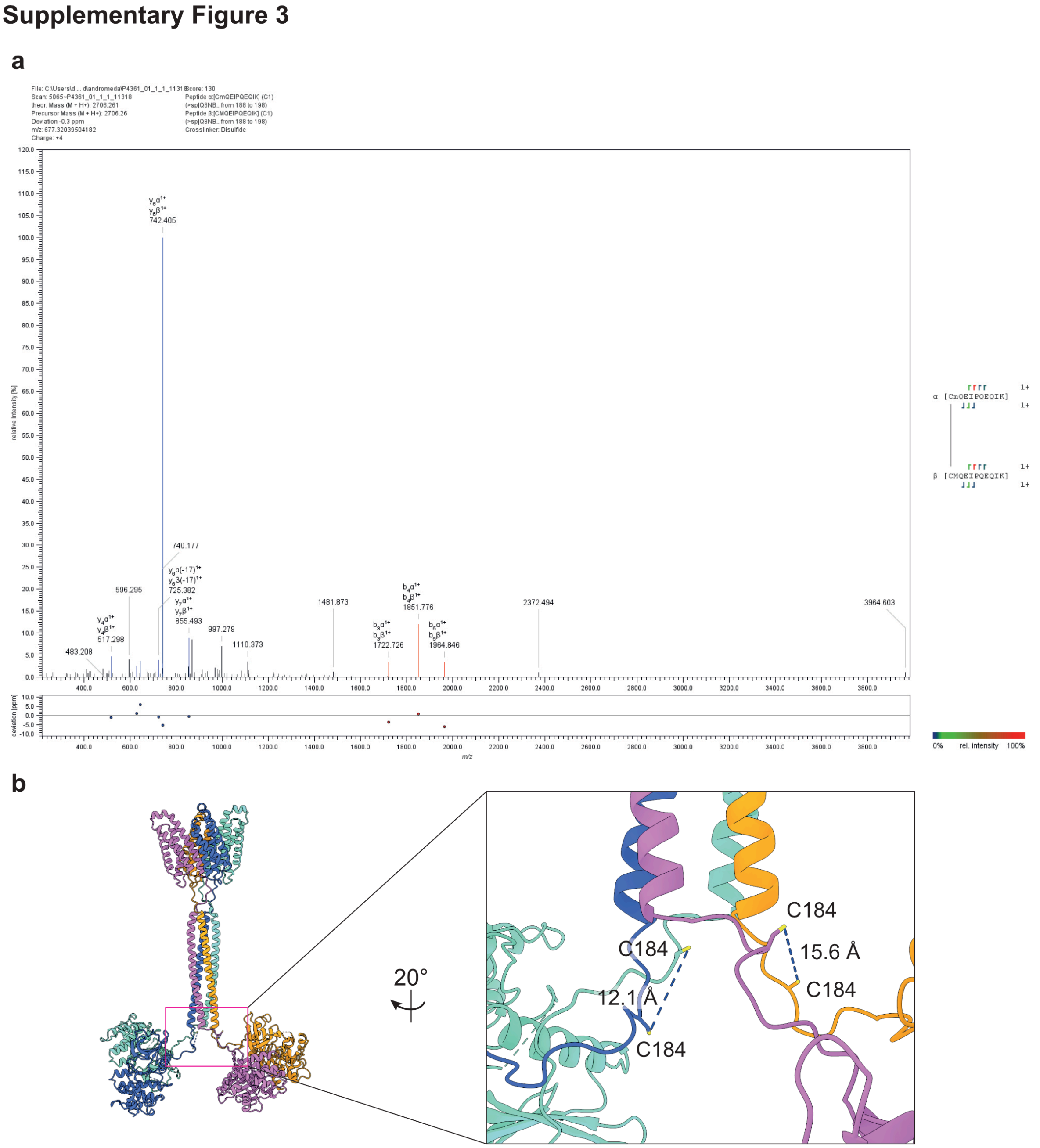

Supplementary Figure 4. The 4HB domains within human MLKL tetramer forms an elaborated bundle with 4-fold rotational symmetry.

a. 2D classes from negative stain electron microscopy on full-length human MLKL tetramer stabilized by Mb27. Number of particles in each class is shown as white text. **b.** The 4HB domain tetramer is stabilized by Q117-mediated hydrogen bonds. *Left*, model of full-length human MLKL tetramer (cartoon; colored by chains). *Middle*, top-down view of the tetrameric arrangement of 4HB domains within the human MLKL tetramer. *Right*, zoom in panel showing the core of 4HB domain tetramer with Q117 side chain shown as sticks. **c**. An alternative human MLKL tetramer model generated by ColabFold (grey cartoon; chain A colored according to pLDDT score). In this model, the 4HB domain is bound by the brace region in an autoinhibited conformation (left), and residues R34, Q117, and Q120 are not involved in intermolecular interactions, which is inconsistent with the observation that mutating these residues disrupts necroptosis.

Supplementary Figure 4

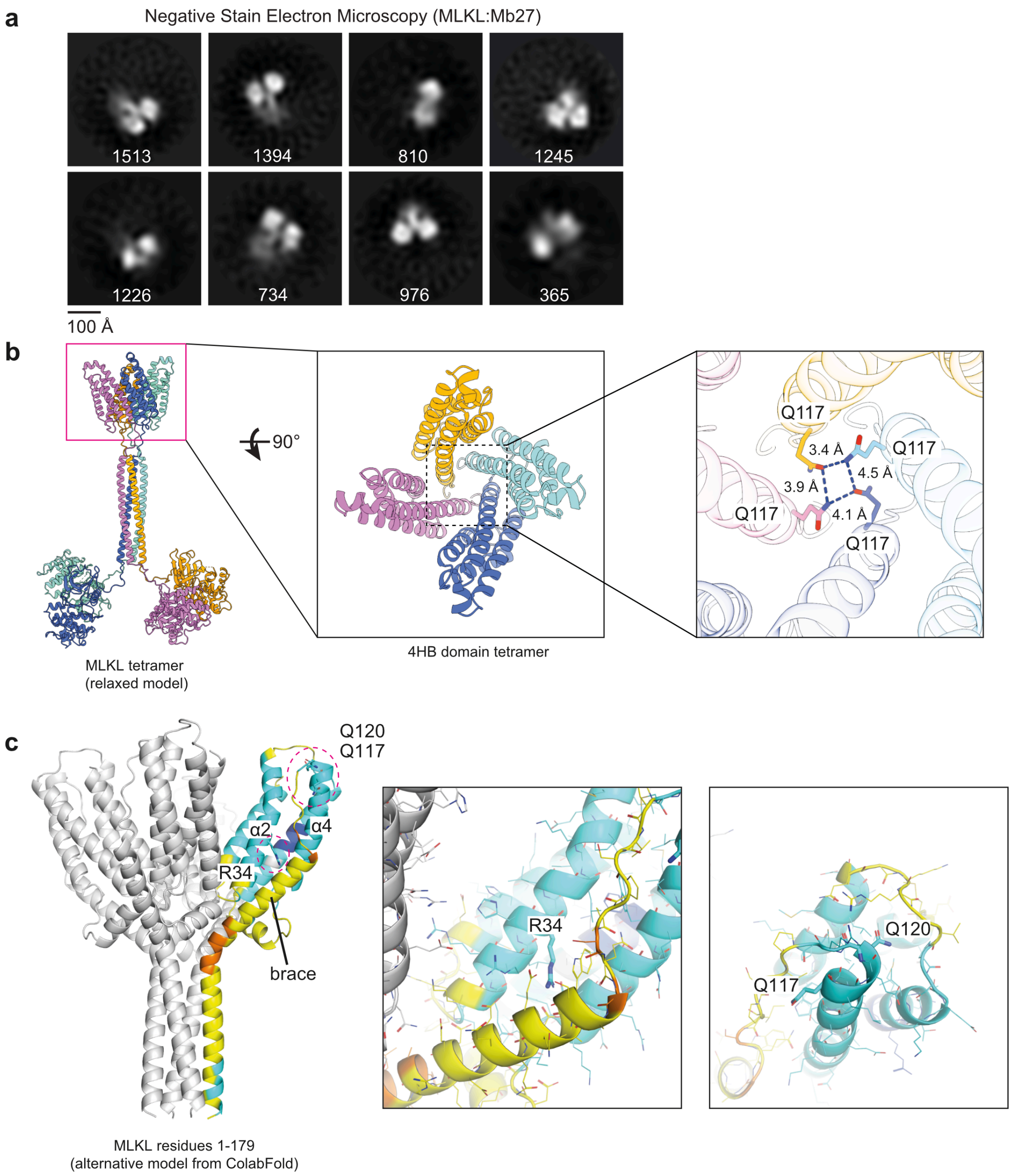

Supplementary Figure 5. Human MLKL tetramerization is essential for membrane disruption and cell death by necroptosis.

a. Expression of recombinant human MLKL 4HB domain (residues 2-123) without the brace region. *Top*, map of expression construct. A HRV 3C protease cleavage site is introduced into a construct encoding an N-terminal TEV-cleavable GST-tagged human MLKL (residues 2-154; 4HB domain plus brace region) construct to replace the flexible loop between the 4HB domain and the brace region (residues 123-130). Recombinant human MLKL 4HB was acquired by cleavage with both TEV and HRV 3C protease. *Left*, size-exclusion chromatograms of human MLKL 4HB domain (black) and human MLKL 4HB domain plus the brace region on a Superdex 75 10/300 GL. Fractions analysed by SDS-PAGE are labelled on the x-axis. *Right*, purified human MLKL 4HB domain analysed by reducing SDS-PAGE. **b**. To induce the expression of wild-type (WT) or mutant human MLKL constructs *MLKL*$^{-/-}$ HT29 cells were stimulated with doxycycline (Dox; overnight) in the presence or absence of necroptotic stimulus (TNF, Smac mimetic, IDN-6556; TSI; 4 h). Protein levels of MLKL, both unphosphorylated and phosphorylated forms, were detected by immunoblotting. Data are representative of triplicate independent experiments. Uncropped blots are shown in Supplementary Figure 7. **c**. The effects of mutation within the tetramerization interface of human MLKL on the kinetics of necroptosis were assessed using IncuCyte SX5 live cell imaging. Stably transduced *MLKL*$^{-/-}$ HT29 cells were treated with doxycycline (Dox; 100 ng/mL) overnight to induce the expression of wild-type (WT) or mutant human MLKL proteins. Cells were then treated with necroptotic stimulus (TSI) and cell death was quantified as a percentage of SYTOX Green positive cells, every hour for 24 h. Cell lines were generated for WT and all MLKL mutants, and assayed in n = 3 independent experiments. Data are plotted as mean ± SEM.

Supplementary Figure 5

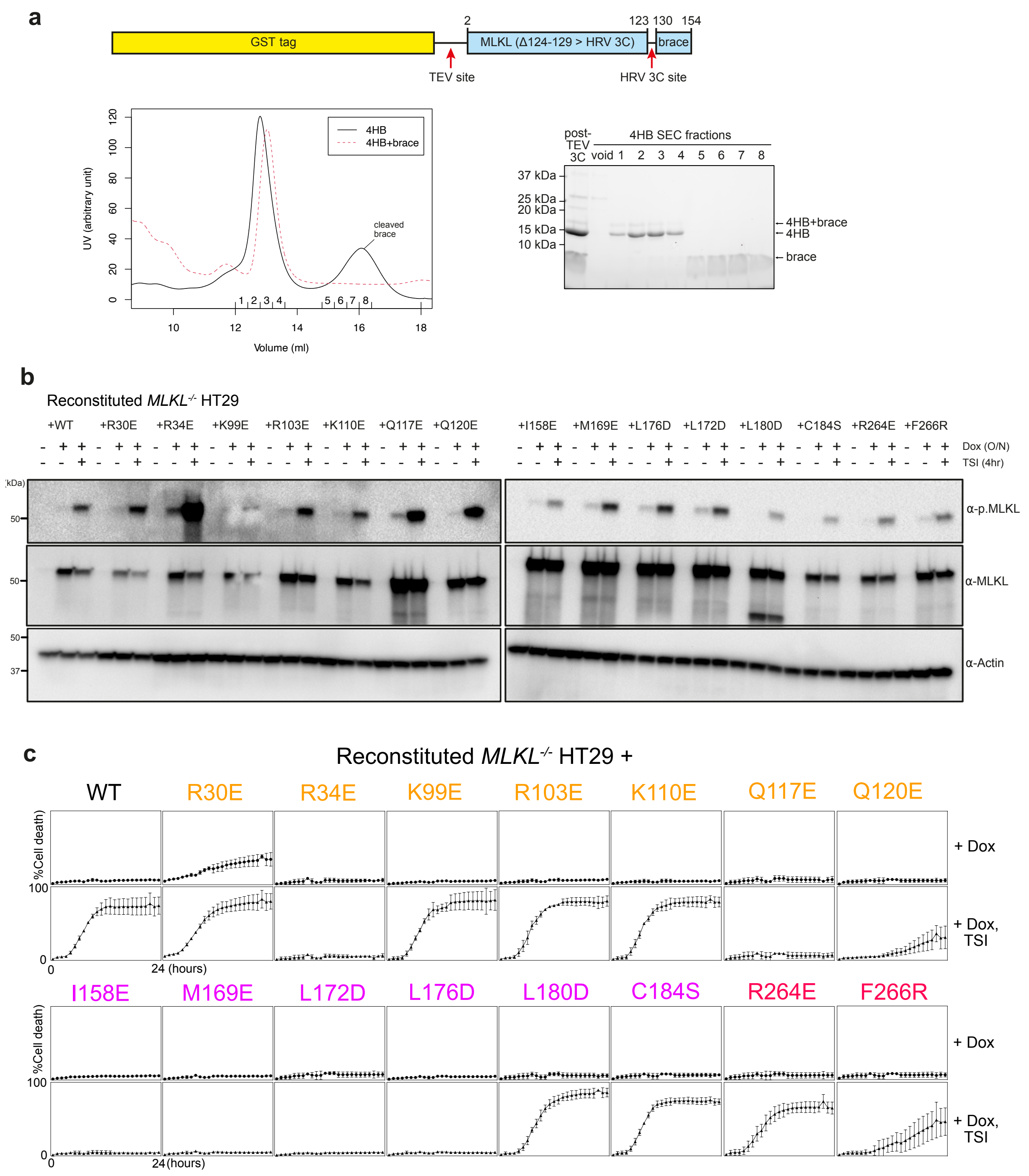

Supplementary Figure 6. Crystallographic lattice and maps of phosphorylated (p)-human MLKL pseudokinase domain.

a. Crystallographic lattice with highlighted asymmetric unit (ASU; drawn in PyMol). Lattice symmetry mates are shown as transparent cartoons, with one ASU highlighted as an opaque cartoon. The αC helix is shown in yellow, and the activation loop in green. **b-c.** Representative density maps ($2F_o$–F_c, 1.52 σ contour); from Coot.

Supplementary Figure 6

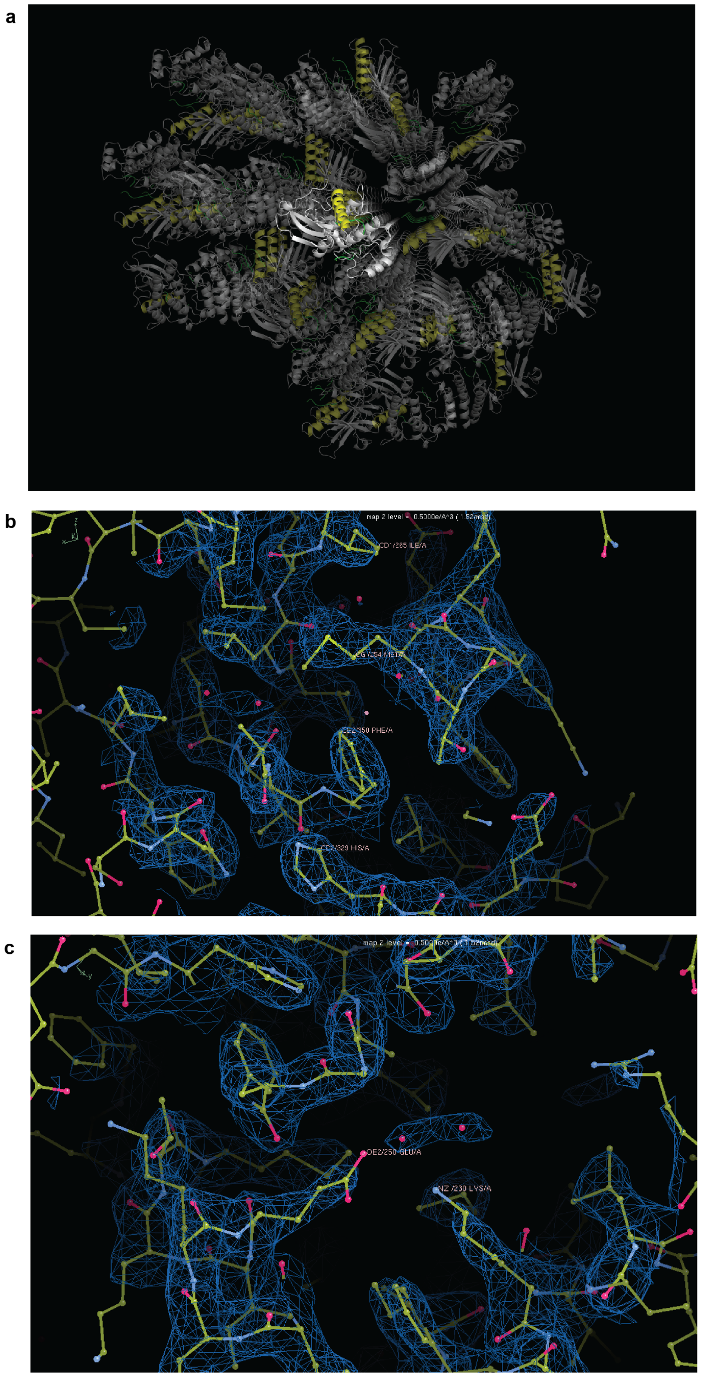

Supplementary Figure 7. Source data for gel images.

The uncropped images correspond to the source data as follows. **a.** Supplementary Figure 5a. **b.** Supplementary Figure 5b. Boxes indicate data presented in Supplementary Figure 5.

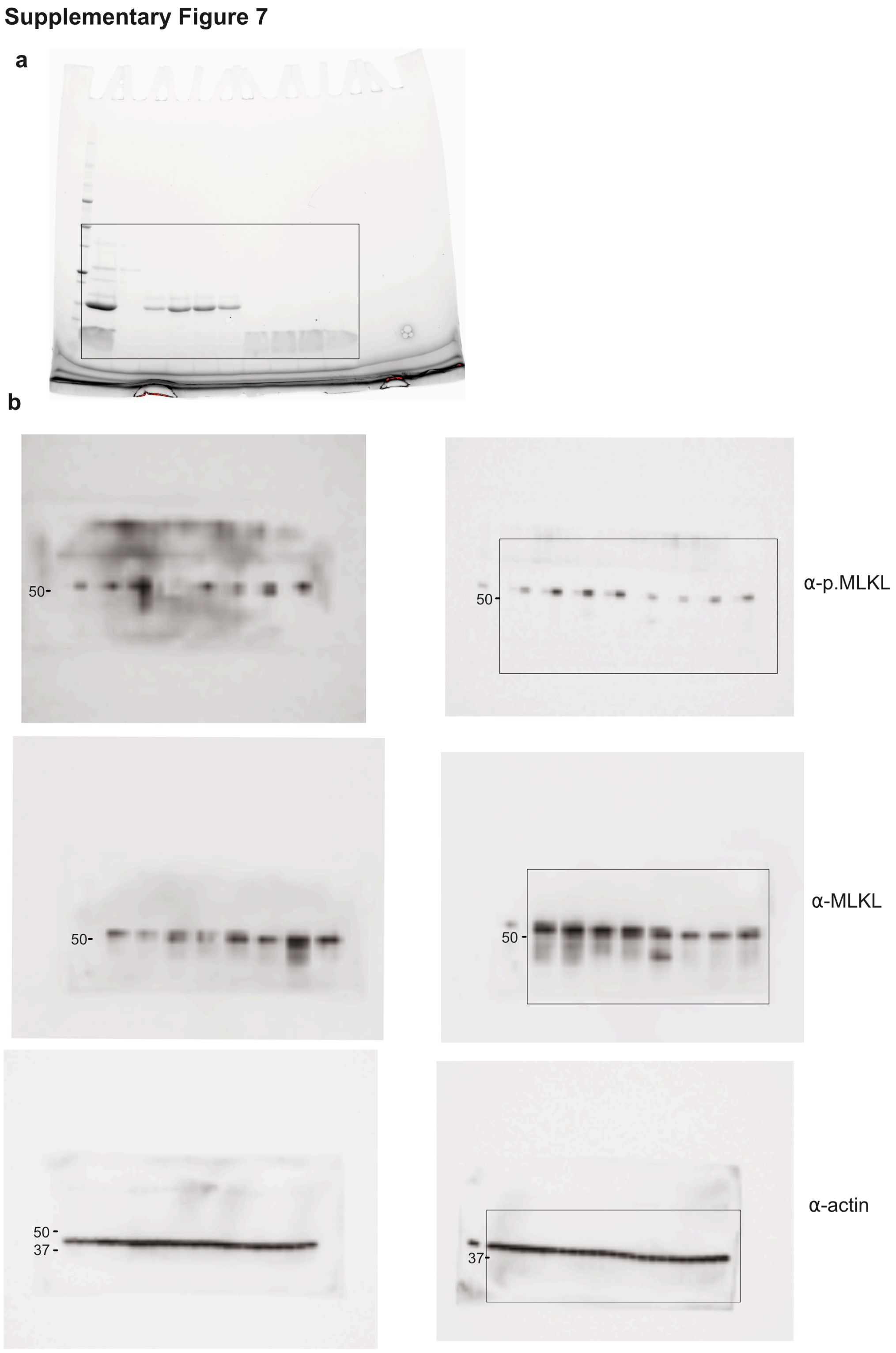

SUPPLEMENTARY REFERENCE

1. Meng, Y., Davies, K.A., Fitzgibbon, C., Young, S.N., Garnish, S.E., Horne, C.R., Luo, C., Garnier, J.-M., Liang, L.-Y., Cowan, A.D., et al. (2021). Human RIPK3 maintains MLKL in an inactive conformation prior to cell death by necroptosis. Nature Communications *12*, 6783. 10.1038/s41467-021-27032-x.
