# Supplementary material for: Phosphorylation-dependent pseudokinase domain dimerization drives full-length MLKL oligomerization
Source: Nat Commun. 2023 Oct 26;14:6804. doi: 10.1038/s41467-023-42255-w (PMC10603135; doi:10.1038/s41467-023-42255-w)
Supplement: Supplementary file 3 — Reporting Summary [file 41467_2023_42255_MOESM3_ESM.pdf]

nature portfolio

Corresponding author(s): Professors James Murphy and Peter Czabotar

Last updated by author(s): Sep 28, 2023

Reporting Summary

Nature Portfolio wishes to improve the reproducibility of the work that we publish. This form provides structure for consistency and transparency in reporting. For further information on Nature Portfolio policies, see our Editorial Policies and the Editorial Policy Checklist.

Statistics

For all statistical analyses, confirm that the following items are present in the figure legend, table legend, main text, or Methods section.

n/a	Confirmed	
☐	☒	The exact sample size (*n*) for each experimental group/condition, given as a discrete number and unit of measurement
☐	☒	A statement on whether measurements were taken from distinct samples or whether the same sample was measured repeatedly
☐	☒	The statistical test(s) used AND whether they are one- or two-sided<br>*Only common tests should be described solely by name; describe more complex techniques in the Methods section.*
☒	☐	A description of all covariates tested
☒	☐	A description of any assumptions or corrections, such as tests of normality and adjustment for multiple comparisons
☐	☒	A full description of the statistical parameters including central tendency (e.g. means) or other basic estimates (e.g. regression coefficient) AND variation (e.g. standard deviation) or associated estimates of uncertainty (e.g. confidence intervals)
☐	☒	For null hypothesis testing, the test statistic (e.g. *F*, *t*, *r*) with confidence intervals, effect sizes, degrees of freedom and *P* value noted<br>*Give P values as exact values whenever suitable.*
☒	☐	For Bayesian analysis, information on the choice of priors and Markov chain Monte Carlo settings
☒	☐	For hierarchical and complex designs, identification of the appropriate level for tests and full reporting of outcomes
☒	☐	Estimates of effect sizes (e.g. Cohen's *d*, Pearson's *r*), indicating how they were calculated

Our web collection on statistics for biologists contains articles on many of the points above.

Software and code

Policy information about availability of computer code

Data collection	IncuCyte S3/SX5 Sartorius Imager Software v2022B, v2021B, v2020C & v2018A.<br>CLARIOstar plate reader 5.70 R2<br>TEM Imaging & Analysis (version 5.0 SP4; Thermo Fisher Scientific), , Relion (3.1.3)<br>ScatterBrain (Linux V 2.71; Australian Synchrotron) was used acquire SAXS data.<br>Rosetta v3.5 was used to optimise AlphaFold models.<br>ColabFold-Multimer (version 2) was used to generate AlphaFold models.
Data analysis	Cell death: IncuCyte S3/SX5 analysis was completed with Sartorius Imager Software v2022B, v2021B, v2020C & v2018A<br>Figure 4c was graphed using Prism v9, with others plotted using R version 4.2.2.<br>Negative stain EM: TEM Imaging & Analysis (version 5.0 SP4; Thermo Fisher Scientific), Relion (3.1.3)<br>Crystallography: XDS (INTEGRATE VERSION Nov 1, 2016 BUILT=20161205), AIMLESS (version 0.7.8), PHASER (2.8.3), COOT (0.9 EL ccp4), Phenix (1.18.2-3874-000), MolProbity (4.02b-467)<br>Visualising protein structures: ChimeraX (1.2.5), PyMOL (version 2.5.0)<br>SAXS: CHROMIXS was used to average SAXS frames and is part of the ATSAS suite. The ATSAS software package (version 3.03) was used to analyze SAXS data.<br>Mass spectrometry: MaxQuant (version 1.6.17.0).

For manuscripts utilizing custom algorithms or software that are central to the research but not yet described in published literature, software must be made available to editors and reviewers. We strongly encourage code deposition in a community repository (e.g. GitHub). See the Nature Portfolio guidelines for submitting code & software for further information.

Data

Policy information about availability of data

All manuscripts must include a data availability statement. This statement should provide the following information, where applicable:

- Accession codes, unique identifiers, or web links for publicly available datasets
- A description of any restrictions on data availability
- For clinical datasets or third party data, please ensure that the statement adheres to our policy

Source data are published as an accompanying file; any additional data, including expression construct sequences, are available from the corresponding authors upon request. Any materials are available from the corresponding authors under Materials Transfer Agreement. The atomic coordinates for the p-MLKL pseudokinase domain crystal structure reported here have been deposited in the Protein Data Bank with the accession number, 8SLZ [http://doi.org/10.2210/pdb8SLZ/pdb]. Previously reported crystal structures depicted in this work are available from the Protein Data Bank with accession numbers: 7MON [http://doi.org/10.2210/pdb7MON/pdb], 7JXU [http://doi.org/10.2210/pdb7JXU/pdb], 7JW7 [http://doi.org/10.2210/pdb7JW7/pdb], 6UX8 [http://doi.org/10.2210/pdb6UX8/pdb]; with 4MWI [http://doi.org/10.2210/pdb4MWI/pdb] used for phasing by molecular replacement.

Research involving human participants, their data, or biological material

Policy information about studies with human participants or human data. See also policy information about sex, gender (identity/presentation), and sexual orientation and race, ethnicity and racism.

Reporting on sex and gender	No human participants used in this study. Commercially-available human-derived cell lines are described below.
Reporting on race, ethnicity, or other socially relevant groupings	N/A
Population characteristics	N/A
Recruitment	N/A
Ethics oversight	N/A

Note that full information on the approval of the study protocol must also be provided in the manuscript.

Field-specific reporting

Please select the one below that is the best fit for your research. If you are not sure, read the appropriate sections before making your selection.

☒ Life sciences ☐ Behavioural & social sciences ☐ Ecological, evolutionary & environmental sciences

For a reference copy of the document with all sections, see nature.com/documents/nr-reporting-summary-flat.pdf

Life sciences study design

All studies must disclose on these points even when the disclosure is negative.

Sample size	Sample sizes are consistent with field-norms. The numbers of independent repeat experiments are stated in figure legends. No statistical calculations were performed prior to these studies. Rather, we selected the samples sizes based on our prior, published cell-based studies, in which robust consistency was evident between assays and n of 3 or more were established as sufficient to reveal any differences between cell lines.
Data exclusions	No data were excluded
Replication	Experimental findings were replicated both through independent experimental repeats (same cell culture used on separate days) and through the use of numerous independent biological replicates (cell lines derived from separate transductions). Exact n are provided in the legends of all figures.
Randomization	Random allocation of cell lines is consistent with field norms. Cells of each independent line were grown as a single pool until immediately before being counted and assigned to experimental groups randomly.
Blinding	Cell lines reconstituted with wild-type and mutant MLKL constructs were assayed in parallel with no assumptions about phenotype, except for the wild-type MLKL lines, which were used as a benchmark to ensure the integrity of data acquisition, the death stimuli and inhibitor controls. While experimenters were not formally blinded to the mutant MLKL construct identities, these were assayed as numerically-coded constructs and the impact of mutation evaluated post-microscopy data acquisition. Consistent data were obtained from independently-generated cell lines expressing equivalent mutant constructs. Studies with recombinant proteins were not blinded; for liposome assays and immunoprecipitations, data were collected for mutant proteins in parallel with wild-type controls without prior expectation of mutant phenotypes.

Reporting for specific materials, systems and methods

We require information from authors about some types of materials, experimental systems and methods used in many studies. Here, indicate whether each material, system or method listed is relevant to your study. If you are not sure if a list item applies to your research, read the appropriate section before selecting a response.

Materials & experimental systems

n/a	Involved in the study
☐	☒ Antibodies
☐	☒ Eukaryotic cell lines
☒	☐ Palaeontology and archaeology
☒	☐ Animals and other organisms
☒	☐ Clinical data
☒	☐ Dual use research of concern
☒	☐ Plants

Methods

n/a	Involved in the study
☒	☐ ChIP-seq
☒	☐ Flow cytometry
☒	☐ MRI-based neuroimaging

Antibodies

Antibodies used

anti-hMLKL, WEHI Clone 7G2 (also commercially available from Merck-Millipore MABC1636) *
anti-FLAG, M2 clone, Sigma- Aldrich; F1804
anti-actin, Sigma-Aldrich A-1978; AC-15
anti-GAPDH, Cell Signalling Technology #2118; 14C10
anti-VDAC, Merck-Millipore, #2450741; AB10527
anti-phospho-hMLKL, Abcam, EPR9514; ab187091*

Validation

* Validation of antibodies via western blot with respective knockout HT29 cells published in Samson et al., 2021 (DOI: 10.1038/s41418-021-00742-x). Specifically anti-hMLKL 7G2 and anti-phospho-hMLKL EPR9514 validated in Fig 1e.

anti-actin, Sigma-Aldrich A-1978; AC-15 - Antibody has been used in publications extensively and validation available at https://www.sigmaaldrich.com/AU/en/product/sigma/a1978, validated for western blot analysis on human foreskin fibroblasts.
anti-FLAG has been validated by the manufacturer (https://www.sigmaaldrich.com/catalog/product/sigma/f1804), and also in our study where no signal was observed prior to induction of exogenous FLAG-tagged Monobodies.
anti-GAPDH, Cell Signalling Technology #2118; 14C10 - Antibody has been used in publications extensively and validation is available at https://www.cellsignal.com/products/primary-antibodies/gapdh-14c10-rabbit-mab/2118, validated for western blot analyses on HeLa, NIH/3T3, C6, HUVEC and L929 cell lines.
anti-VDAC, Merck-Millipore, #2450741; AB10527 - Antibody has been used in publications extensively and validation available at https://www.merckmillipore.com/INTERSHOP/web/WFS/Merck-INTL-Site/en_US/-/USD/ShowDocument-File?ProductSKU=MM_NF-AB10527&DocumentId=null&DocumentType=COA&Language=EN&Country=US&ProductBatchNo=2512107&Origin=PDP, validated for western blot analyses on HepG2 cell lysates.

Eukaryotic cell lines

Policy information about cell lines and Sex and Gender in Research

Cell line source(s)

Unmodified HT29 cells were sourced from the laboratory of John Silke and originate from ATCC. CRISPR-edited derivative HT29 cells lacking MLKL were reported previously (Petrie et al., Nature Commun 2018) and were produced in-house from parental HT29 cells. HEK293T cells were sourced from the laboratory of John Silke, and were originally purchased from the ATCC. Sf21 insect cells were sourced from Merck.

Authentication

HT29 cells were not formally authenticated. However, their morphologies are consistent with the stated cell type. The lack of responsiveness of MLKL-deficient human HT29 cells to necroptotic stimuli and the lack of respective MLKL reactivity by western blot are consistent with the absence of MLKL. HEK293T cells were not formally authenticated, but possessed morphologies consistent with HEK293T cells and successfully generated lentiviruses for subsequent HT29 infection. Sf21 cells were successfully propagated in serum-free insect cell media and successfully transfected with bacmid to generate baculovirus, and subsequently infected to propagate viral stocks and express protein.

Mycoplasma contamination

All mammalian cell lines used were monitored for mycoplasma and were found to be negative in our routine PCR screening.

Commonly misidentified lines
(See ICLAC register)

Nil
